# Starting a Dental Practice*This is an extract from a paper published in *Oral Hygiene* for September, about the difficulties a young graduate encountered in getting located.

**Published:** 1919-10

**Authors:** 


### STARTING A DENTAL PRACTICE *

Realizing that I wasn't getting anywhere, I went out to call on a friend who runs a hardware store up town, and took up the matter with him.

"When are you going to open your tooth palace?" he asked jocularly.

I told him my difficulties, my perplexities and uncertainties.

"What's the matter with this neighborhood?" he wanted to know.

I told him it didn't seem to contain the class of people that would require the best dental work.

"Well, what's your idea in practicing dentistry, anyway?" he exclaimed.

I told him that we were pledged to the service of humanity.

"Some humanity around here, ain't there?" he asked. "Don't they have holes in their teeth that need plugging? Don't they have toothache? Bet you could keep busy ten hours a day pulling stumps. And plates—I'll wager there

* This is an extract from a paper published in *Oral Hygiene* for September, about the difficulties a young graduate encountered in getting located.

are a thousand needed within a dozen squares of here right now.''

''But,'' I said, ''dentists usually locate in the more exclusive portions of the city or in professional buildings; they desire a selected class of patients who seek them out and come to them.''

''Then your 'service to humanity' is all a bluff,'' he said. ''You want work and here it is. These people are willing to pay for it and to treat you as a neighbor and a friend. They are the kind of people that keep the grocers, the doctors, and the department stores busy. But *you* don't want them. You want a 'selected' class. These people are as good as you are, and better off than you are, and yet you are running away from them.''

''But don't a lot of these people patronize the advertising dentists?'' I asked.

''Yes,'' he said, ''they do, and I suppose they will keep on doing it as long as you 'ethical' fellows run away from them. You don't want this sort of practice, and yet you denounce the fellow who comes in and gets it. If you don't approve of advertising, why don't you show that this work can be done without advertising?''

I had no answer; here was a new angle that neither the professors in the college nor my dealer had touched upon.

My friend went right on: ''You fellows make me sick. I read the other day about all kinds of disease being the result of bad teeth, and every toothpaste ad says 'visit your dentist periodically,' and yet the dentists hide from us. Do you think that if you settle in an exclusive locality I'll send patients to you? No, I'll patronize home industries and tell people to go to the advertising man who is human enough to know that people of our kind exist.''

He saw that I was beginning to waver, so he pressed his advantage.

''You can get an office next door,'' he said. ''Take it tomorrow. I'll back you for the rent. I guess you haven't much to start on, but I'll help you out. Go buy the things you need to work with, but nothing for show. Pay cash. Furnish your office like your mother furnishes her parlor; it seems that simple things are fashionable now. Make your patients feel at home and not as if they were in a vault or a corridor of the Waldorf-Astoria.''

''By George!'' I exclaimed. ''This sounds like sense.''

''It is sense,'' he rejoined. ''Be human. A doctor is allowed to practice because the people need him, and it's the only excuse a dentist has. It is the sensible thing, then, to go direct to the people, live with them, know them, serve them, and trust them to do the rest.''

''I wonder what the advertising man around the corner will do?'' I mused.

''Well,'' said my friend, ''I think the best will survive, and you may be sure he will stay until something better replaces him. Say—I've got an idea!'' he finished.

''Shoot it,'' I told him, for now he had me sold on the proposition.

''It's this. You settle next door and hang out your sign. You don't advertise and the other fellow does. The people will notice the difference and will get the idea quickly. You see, you'll get a lot of advertising out of *not advertising.*''

Well, all of that was seven years ago. I haven't made a fortune, but I have been busy. I have paid all my debts and now own the house in which my office is located. Many of the people around call me ''Doc,'' and I rather like it, for it signifies that I belong to the community. The advertising man is still around the corner, but my practice is worth more than his and I think I may say modestly that mine represents the cream.

I am thinking seriously of recommending to my college that they give a course on ''How to get into practice and how to conduct it,'' and I shall suggest my old hardware friend as the professor in charge.

